# Cerebral microbleeds in adult survivors of childhood acute lymphoblastic leukemia treated with cranial radiation

**DOI:** 10.1038/s41598-020-57682-8

**Published:** 2020-01-20

**Authors:** Nicholas S. Phillips, Claudia M. Hillenbrand, Bogdan G. Mitrea, Jason Yan, Chenghong Li, Matthew A. Scoggins, Thomas E. Merchant, Gregory T. Armstrong, Deokumar Srivastava, Ching-Hon Pui, Leslie L. Robison, Melissa M. Hudson, Kevin R. Krull, Noah D. Sabin

**Affiliations:** 1 Department of Epidemiology and Cancer Control, Memphis, TN USA; 2 Department of Diagnostic Imaging, Memphis, TN USA; 3 Department of Biostatistics, Memphis, TN USA; 4 Department of Radiation Oncology, Memphis, TN USA; 5 Department of Oncology, Memphis, TN USA; 6 Department of Psychology St. Jude Children’s Research Hospital, Memphis, TN USA; 7 Department of Psychiatry and Behavioral Medicine, Wake Forest Baptist Medical Center, Winston-Salem, NC USA

**Keywords:** Cognitive neuroscience, Magnetic resonance imaging

## Abstract

Cranial radiation therapy is associated with white matter-specific brain injury, cortical volume loss, mineralization, microangiopathy and neurocognitive impairment in survivors of childhood acute lymphoblastic leukemia. In this retrospective cross-sectional analysis, neurocognitive testing and 3 T brain MRI’s were obtained in 101 survivors treated with cranial radiation. Small focal intracerebral hemorrhages only visible on exquisitely sensitive MRI sequences were identified and localized using susceptibility weighted imaging. Modified Poisson regression was used to assess the effect of cranial radiation on cumulative number and location of microbleeds in each brain region, and multiple linear regression was used to evaluate microbleeds on neurocognitive outcomes, adjusting for age at diagnosis and sex. At least one microbleed was present in 85% of survivors, occurring more frequently in frontal lobes. Radiation dose of 24 Gy conveyed a 5-fold greater risk (95% CI 2.57–10.32) of having multiple microbleeds compared to a dose of 18 Gy. No significant difference was found in neurocognitive scores with either the absence or presence of microbleeds or their location. Greater prevalence of microbleeds in our study compared to prior reports is likely related to longer time since treatment, better sensitivity of SWI for detection of microbleeds and the use of a 3 T MRI platform.

## Introduction

Cranial radiation therapy (CRT) has been associated with cognitive impairment in survivors of childhood acute lymphoblastic leukemia (ALL)^1,2^. Adult survivors of childhood ALL treated with 24 Gy CRT were six times more likely to develop cognitive impairments than survivors treated with chemotherapy only in one large cohort study^3^. Radiation therapy has been associated with structural changes in the brain including white matter injury, cortical volume loss, mineralization, and microangiopathy^4,5^, which in turn were related to neurocognitive impairments^6^.

The incidence of microbleeds in the general population is between 5–35% with large variance reflecting increasing prevalence with age^7,8^. In a study using early susceptibility imaging techniques, Chan and colleagues reported microbleeds in 55% of 40 leukemia survivors treated with CRT at an average duration of 12.2 years from diagnosis^9^. To our knowledge, there are no studies examining the associations between microbleeds and neurocognitive function in long-term survivors of childhood ALL. However, in a multi-institutional cohort of 149 pediatric brain tumor survivors, those who had CRT had a cumulative incidence of microbleeds of 48.8% at 5 years; in this series the presence of microbleeds in frontal lobes was associated with worse executive function and in temporal lobes with poorer verbal memory^10^. Moreover, in a longitudinal study of 959 Chinese volunteers without cancer who were 50 years of age or older, lobar microbleeds, but not deep or infratentorial microbleeds, were associated with impaired visuospatial executive function using high field (3 T) susceptibility weighted imaging (SWI)^11^.

The aims of the current study were to evaluate survivors of childhood ALL during adulthood using 3 T MRI and a well-established high quality microbleed sensitive sequence to examine if relatively low doses of cranial radiation were associated with the development of microbleeds, and to investigate if the presence, frequency and/or location of microbleeds was associated with neurocognitive impairment.

## Results

Of the 101 survivors of ALL included in this study (55 females, mean [range] time since diagnosis 27.6 [19.18–46.01] years), 64 (63.4%) had ≤18 Gy cranial irradiation. High-dose intravenous methotrexate was administered to 49.5% and intrathecal methotrexate to 97% of the survivors. Most survivors were college graduates or had taken classes at the college level (67.4%) with only 9.2% having not completed high school. Most survivors were full-time employed (76.0%) with only 17.7% unemployed at the time of testing (Table 1).

**Table 1:** Demographic and treatment characteristics of childhood cancer survivors.

Characteristic	Subgroup	N (%)
Biological sex	Female	55 (54.5)
	Male	46 (45.5)
Race	Black	8 (7.9)
	Other	2 (2.0)
	White	91 (90.1)
Cranial radiation dose (Gy)	18	64 (63.4)
	24	37 (36.6)
Radiation source type	Cobalt 60	27 (26.7)
	Linear Acceleration	74 (73.3)
Radiation site	Cranio-Spinal	1 (1.0)
	Cranium	99 (98.0)
	Total Body Irradiation	1 (1.0)
High dose MTX use	No	51 (50.5)
	Yes	50 (49.5)
Intrathecal MTX use	No	3 (3.0)
	Yes	98 (97.0)
Education	Unknown	3
	<High school	9 (9.2)
	High school/GED	23 (23.5)
	Some college	32 (32.7)
	>=College graduate	34 (34.7)
Employment	Unknown	5
	Unemployed	17 (17.7)
	Part time	6 (6.3)
	Full time	73 (76.0)

Eighty-five percent (N = 86) of survivors in this study had one or more microbleeds. Microbleeds occurred more frequently in the frontal lobes, followed by temporal lobes, the parietal/occipital lobes, sub-lobar/midbrain and finally the cerebellum, pons and brainstem (Table 2). No significant difference in the mean number of microbleeds was found between white matter and grey matter (N = 64, SE 0.358, p = 0.053).

**Table 2:** Number of microbleeds by anatomic location, and grey or white matter region.

Location of microbleeds	Mean	Std. Dev	Median	Min	Max
Frontal	1.7	4.3	1	0	40
Temporal/Limbic	1.7	2.9	1	0	20
Occipital/Parietal	1.1	2.5	0	0	17
Cerebellum/Brainstem	0.6	1.1	0	0	7
Midbrain/Sub-lobar^a^	0.8	1.2	0	0	16
White matter	1.7	2.5	1	0	16
Grey matter	1.0	1.4	1	0	7
Cumulative count	6.0	10.2	3	0	81

^a^Sub-lobar denotes the region filling the remaining undefined volume within each hemisphere.

A CRT dose of 24 Gy conveyed a 5-fold greater risk (RR 5.15, 95% CI 2.57–10.32) of having 6 or more microbleeds than 18 Gy (Table 3). A CRT dose of 24 Gy conveyed a 2-fold greater risk than 18 Gy for the presence of frontal lobe microbleeds (relative risk [RR] 1.92, 95% confidence interval [95%CI] 1.41–2.61) and temporal lobe microbleeds (RR 2.12, 95% CI 1.52–2.95), and a nearly 3-fold greater relative risk for an occipital/parietal lobe microbleeds (RR 2.73, 95% CI 1.72–4.31). We found no significant increased risk for microbleeds with increasing time since diagnosis (RR 1.1, 95% CI 0.98–1.23), cumulative vincristine (RR 0.98, 95% CI 0.96–1.00) or cumulative asparaginase dose (RR 0.99, 95% CI 0.94–1.04).

**Table 3:** Relative Risk of microbleed by location and radiation dose, adjusting for age at diagnosis and sex.

Presence of microbleeds	levels	Relative Risk (95% CI)	P-value
Frontal lobe	24 Gy vs 18 Gy	1.92 (1.41, 2.61)	<0.001
Limbic/Temporal lobe	24 Gy vs 18 Gy	2.12 (1.52, 2.95)	<0.001
Occipital/Parietal lobes	24 Gy vs 18 Gy	2.73 (1.72, 4.31)	<0.001
Anterior/posterior/Medulla, Pons	24 Gy vs 18 Gy	1.95 (1.18, 3.22)	0.013
Sub-lobar^a^/Midbrain	24 Gy vs 18 Gy	2.24 (1.43, 3.52)	0.001
Top tertile of total number of microbleeds (6 or more)	24 Gy vs 18 Gy	5.15 (2.57, 10.32)	<0.001

^a^Sub-lobar denotes the region filling the remaining undefined volume within each hemisphere.

Survivors demonstrated worse performance on 12 neurocognitive outcomes and two self-report outcomes compared to population norms, and these outcomes were selected for subsequent analysis (Table 4). Neurocognitive scores and self-reported outcomes were not associated with CRT dose after adjusting for multiple comparisons (Table 5). No associations were found with location of microbleeds and neurocognitive testing (Table 6). An exploratory analysis found that occipital/parietal lobe microbleeds were associated with self-reported problems in shifting between tasks (SE 0.24; p = 0.031) and working memory (SE 0.27; p = 0.033), while cerebellum and brainstem microbleeds were associated with self-reported working memory problems (SE 0.27; p = 0.005). The total number of microbleeds was not significantly associated with any of the selected neurocognitive tasks, adjusting for age at diagnosis and sex.

**Table 4:** Neurocognitive outcomes among adult survivors of childhood ALL compared to national norms.

Neurocognitive test	Mean (95% CL)	FDR p-value
**Intelligence**		
Verbal ability	−0.65 (−0.90, −0.4,)	<0.001
Perceptual ability	−0.04 (−0.23, 0.16)	0.79
**Processing speed**		
Dominant motor speed	−0.76 (−0.96, −0.53)	<0.001
Non-dominant motor speed	−0.78 (−1.0, −0.53)	<0.001
Visual-motor speed	−0.39 (−0.56, −0.23)	<0.001
Visual speed	−0.20 (−0.39, −0.00)	0.10
Processing speed	−0.36 (−0.56, −0.17)	0.001
Reaction time	0.21 (−0.03, 0.44)	0.17
**Executive Function**		
Cognitive flexibility	−0.79 (−1.10, −0.48)	<0.001
Working memory	−0.48 (−0.68, −0.27)	<0.001
**Attention**		
Focused attention	−0.14 (−0.39, 0.12)	0.43
Omissions	−0.05 (−0.32, 0.23)	0.79
Commissions	−0.10 (−0.34, 0.14)	0.50
Variability	−0.11 (−0.36, 0.14)	0.50
Detectability	−0.087 (−0.30, 0.12)	0.50
**Memory**		
Memory span	−0.57 (−0.79, −0.35)	<0.001
New learning	−0.16 (−0.41, 0.085)	0.31
Short-term recall	0.00 (−0.23, 0.23)	1.00
Long-term recall	−0.066 (−0.30, 0.17)	0.68
**Academics**		
Reading	−0.44 (−0.54, −0.34)	<0.001
Math	−0.73 (−0.92, −0.54)	<0.001
**BRIEF Self-Report**^**a**^		
Inhibitory control	0.12 (−0.07, 0.32)	0.32
Behavioral flexibility	0.40 (0.16, 0.63)	0.003
Self-monitoring	−0.025 (−0.25, 0.20)	0.85
Self-initiation	0.14 (−0.071, 0.36)	0.31
Working memory	0.90 (0.64, 1.16)	<0.001
Planning	0.15 (−0.067, 0.37)	0.31
Task completion	0.22 (0.003, 0.43)	0.10
Organization	0.075 (−0.105, 0.26)	0.50

^a^Denotes that higher scores for the self-report indicates worse outcomes.

**Table 5:** Neuropsychological outcomes by cranial radiation dose group.

Neurocognitive Outcome	<=18 Gy Mean (95% CI)	>=24 Gy mean (95%CI)	P-value
Verbal ability	−0.669 (−1.005, −0.332)	−0.632 (−0.989, −0.276)	0.889
Cognitive flexibility	−0.726 (−1.121, −0.331)	−0.899 (−1.407, −0.391)	0.593
Dominant motor speed	−0.731 (−1.017, −0.446)	−0.802 (−1.199, −0.405)	0.769
Non-dom motor speed	−0.726 (−1.039, −0.413)	−0.877 (−1.319, −0.436)	0.567
Memory span	−0.591 (−0.890, −0.291)	−0.535 (−0.849, −0.221)	0.810
Working memory	−0.441 (−0.727, −0.154)	−0.542 (−0.825, −0.260)	0.639
Visual-Motor speed	−0.401 (−0.625, −0.177)	−0.378 (−0.622, −0.135)	0.896
Processing speed	−0.341 (−0.607, −0.074)	−0.398 (−0.672, −0.125)	0.779
Reading	−0.404 (−0.532, −0.277)	−0.489 (−0.657, −0.321)	0.421
Math	−0.702 (−0.953, −0.450)	−0.778 (−1.054, −0.502)	0.696
**BRIEF Self-report**^**a**^			
Behavioral flexibility	0.298 (0.025, 0.572)	0.567 (0.122, 1.012)	0.277
Working Memory	0.844 (0.530, 1.159)	1.008 (0.531, 1.486)	0.551

^a^Denotes that higher scores for the self-report indicates worse outcomes.

**Table 6:** Neuropsychological testing outcomes by location of microbleeds, adjusting for age at diagnosis and sex.

Neurocognitive Outcome	Frontal lobe		Limbic/Temporal lobe		Occipital/Parietal lobe		Cerebellum/Brainstem		Sub-lobar/Midbrain	
	Est (SE)	P	Est (SE)	P	Est (SE)	P	Est (SE)	P	Est (SE)	P
**Location of microbleed**										
Verbal ability	0.21 (0.26)	0.42	0.38 (0.26)	0.14	0.07 (0.26)	0.80	0.26 (0.26)	0.33	0.13 (0.26)	0.61
Cognitive flexibility	0.10 (0.32)	0.77	0.21 (0.32)	0.52	0.03 (0.32)	0.93	−0.09 (0.33)	0.78	0.23 (0.32)	0.48
Dominant motor speed	−0.09 (0.24)	0.72	0.11 (0.24)	0.65	−0.05 (0.24)	0.84	0.10 (0.24)	0.69	−0.25 (0.24)	0.31
Non-Dom motor speed	−0.24 (0.27)	0.37	0.09 (0.26)	0.72	−0.33 (0.26)	0.22	−0.06 (0.27)	0.81	−0.19 (0.27)	0.49
Memory span	−0.11 (0.23)	0.65	0.17 (0.23)	0.47	0.17 (0.23)	0.47	0.09 (0.23)	0.70	0.19 (0.23)	0.41
Working memory	−0.05 (0.22)	0.83	0.11 (0.21)	0.60	0.23 (0.21)	0.28	0.15 (0.22)	0.48	0.21 (0.22)	0.34
Visual-motor speed	−0.11 (0.17)	0.51	−0.04 (0.17)	0.81	0.01 (0.17)	0.93	−0.27 (0.17)	0.11	−0.17 (0.17)	0.33
Processing Speed	−0.22 (0.20)	0.29	−0.21 (0.20)	0.29	−0.04 (0.20)	0.84	−0.32 (0.20)	0.12	−0.31 (0.20)	0.12
Reading	−0.04 (0.11)	0.71	−0.10 (0.10)	0.33	−0.13 (0.10)	0.22	0.08 (0.11)	0.46	0.01 (0.11)	0.91
Math	0.05 (0.19)	0.78	0.17 (0.19)	0.36	−0.07 (0.19)	0.72	−0.05 (0.19)	0.80	0.01 (0.19)	0.94
**BRIEF Self-report**^**a**^										
Behavioral flexibility	0.07 (0.25)	0.77	0.34 (0.24)	0.16	0.53 (0.24)	0.031	0.39 (0.25)	0.12	0.32 (0.25)	0.20
Working memory	−0.01 (0.28)	0.97	0.14 (0.27)	0.61	0.58 (0.27)	0.033	0.77 (0.27)	0.005	0.12 (0.28)	0.68

^a^Denotes that higher scores for the self-report indicates worse outcomes.

To further explore associations between cerebral microbleeds and cognitive impairment, we conducted sensitivity analyses comparing 15 survivors with no microbleeds to the 15 survivors with 10+ microbleeds (Table 7). No difference was found in the neurocognitive scores between those two groups. Moreover, no association was found between the number, duration or severity of hypertension and microbleed count and no association was found between high-dose methotrexate, intrathecal methotrexate and microbleed count. Therefore, methotrexate exposure was excluded in the final analysis.

**Table 7:** Exploratory univariate analysis comparing 15 survivors with no microbleed to the 15 survivors with 10+ microbleed.

Neurocognitive outcome	No MB Mean (95% CL)	10 + MBs mean (95%CL)	P-value
Verbal ability	−0.993 (−1.901, −0.085)	−0.447 (−1.105, 0.212)	0.305
Cognitive flexibility	−1.111 (−2.371, 0.149)	−0.702 (−1.616, 0.212)	0.578
Dominant Motor speed	−0.818 (−1.568, −0.068)	−0.782 (−1.457, −0.108)	0.940
Non-dom Motor speed	−0.667 (−1.507, 0.173)	−0.858 (−1.624, −0.091)	0.721
Memory span	−0.240 (−0.759, 0.279)	−0.333 (−0.810, 0.144)	0.779
Working memory	−0.484 (−0.915, −0.054)	−0.360 (−0.906, 0.186)	0.704
Visual-motor speed	−0.400 (−0.914, 0.114)	−0.400 (−0.750, −0.050)	1.000
Processing speed	−0.284 (−0.857, 0.288)	−0.543 (−0.914, −0.172)	0.430
Reading	−0.357 (−0.541, −0.174)	−0.419 (−0.682, −0.156)	0.680
Math	−0.748 (−1.442, −0.053)	−0.695 (−1.132, −0.258)	0.891
**BRIEF self-report**^**a**^			
Behavioral flexibility	0.257 (−0.344, 0.858)	0.464 (−0.351, 1.279)	0.662
Working Memory	0.614 (−0.161, 1.390)	0.914 (0.019, 1.810)	0.589

^a^Denotes that higher scores for the self-report indicates worse outcomes.

## Discussion

This study used 3 T MRI and SWI imaging to demonstrate that the incidence of cerebral microbleeds in survivors of ALL, 19 years or more from diagnosis, is greater than previously described. Microbleeds were present in 85% of our survivors compared to only 55–57% in the previous reports^9,12^. The greater incidence of microbleed’s reported in our study is likely related to the greater time since diagnosis and treatment, the greater sensitivity of SWI for detection of microbleeds and the use of a 3 T MRI platform for our study. Prior investigations included 1.5 T MRI and T2* GRE sequences and as such had lower sensitivity for smaller microbleeds^13^. Similar to our study, Neu *et al*., using SWI on a 3 T MRI platform, found that 36 of 40 (90%) of brain tumor survivors treated with cranial radiation had microbleeds at a mean of 13.5 years after diagnosis^14^ and the total number of microbleeds correlated with greater whole brain radiation dose and time since diagnosis. A recent study of 113 adult brain tumor patients treated with cranial radiation who received serial SWI’s at 7 T were found to have an 18% increase in volume and 11% increase in number of microbleeds per year^15^.

Morrison *et al*., however, found no association between maximum radiation dose to the brain and microbleed development in adult brain tumor patients^15^. Similarly, in a previous study of 90 children treated with cranial radiation for a mixture of CNS tumors and leukemias, no significant difference in cerebrovascular abnormalities were found between patients treated with low (18 Gy) or high (at least 32 Gy) doses of CRT^4^. In contrast to these studies we found that higher CRT doses (24 Gy vs 18 Gy) conveyed a greater risk for the microbleed development. The difference between our findings and those of Morrison *et al*. may be related to our survivors’ exposure to radiation as children as opposed to adulthood in the Morrison study. Interestingly, Morris *et al*. found that multiple neurosurgical resections conveyed a greater risk for the development of microbleeds^15^. This may also explain the differences seen between brain tumor and ALL survivors. Additionally, the participants in the Koike *et al*. study were only evaluated 10 years or less from time of diagnosis while the survivors in our cohort were imaged between 19 to 46 years after diagnosis. With a greater time since treatment, microbleeds may differentially develop based on cranial radiation dose. Cranial radiation induced microbleeds have been shown to be produced by the release of vEGF induced by radiation exposure and develop as early as three months after exposure and can continue to develop over decades^16,17^. In the context of the previous studies, our findings would suggest that the natural progression of radiation induced microbleeds in childhood ALL survivors is to increase in number with age and that CRT dose may be related to the amount of increase.

We found no evidence that the increased number or location of the microbleeds was associated with neurocognitive test outcomes in survivors. There is limited data in the literature regarding the association between microbleeds in childhood ALL survivors and neurocognitive outcomes. In the only other study of ALL survivors to investigate possible neurologic symptoms associated with microbleeds, only two of 43 patients with focal susceptibilities on MRI presented with neurologic manifestations^12^. One was associated with a meningioma related to the patient’s clinical presentation and the other had a microbleed in the frontal lobe that was not correlated with the presenting clinical symptoms. This supports our finding that there is no relationship between number or location of microbleeds and neurocognitive outcomes. In addition, we found no difference in cognitive outcomes between survivors who had received 18 and 24 Gy although both groups demonstrated significantly lower neurocognitive performance compared to age adjusted norms.

A prior study of pediatric brain tumor survivors found microbleeds were associated with lower executive function and verbal memory scores^10^. Similar to the Chan *et al*. and Faraci *et al*. investigations in ALL patients, the Roddy *et al*. study did not use the most sensitive technique for microbleed detection (SWI) in all subjects. Additionally, some imaging was performed on less sensitive 1.5 T MRI platforms which could have led to under detection of microbleeds^18^. Further, it is possible that the nonuniform MRI parameters used in the Roddy *et al*. study led to undercounting of smaller cerebral microbleeds, which introduced non-random bias in their data and affected detection of microbleed associations with neurocognitive measures. Our results corroborate those found in a study of adults with mild cognitive impairment and cerebral microbleeds, conducted using 3 T MRI and SWI sequences, which found no significant associations between cognitive decline and the number or location of the microbleeds^8^.

Interestingly, survivors self-reported difficulties with mentally shifting between tasks and working memory problems were associated with microbleeds in the occipital/parietal lobe and cerebellum, midbrain and pons. There are limited data in the literature looking at the association between self-reported outcomes and microbleeds in any population. One possible explanation is that this association could be the result of lesions in the parietal lobe impacting survivor’s self-perception or self-image^19^. However, our study could be limited in that the neurocognitive domains tested were not adequate to capture the cognitive problems self-reported by the survivors or that these were spurious results. Additionally, although we did not find any association between hypertension and the number of microbleeds, we cannot exclude that additional comorbidities associated with aging may have greater impact on cognitive deficits in the aging survivor cohort than microbleeds. It is possible that the size of the microbleeds might influence neurocognitive function. However, the physics of susceptibility imaging limits our ability to estimate the size of the microhemorrhage, as the susceptibility field only represents the concentration of susceptibility inducing constituents and not necessarily the spatial distribution of those components^20^.

In adult survivors of childhood ALL, routine assessment of radiation induced cerebral microbleeds may not be warranted to monitor their neurocognitive impact. Our data would indicate that although the number of microbleeds increases with age, lifestyle and chronic aging conditions may contribute more to cognitive decline in this population than cumulative microbleed burden. A prospective longitudinal study would be needed to determine if survivors suffer cognitive declines over time and if those declines correlate better with chronic health conditions or microbleed accumulation or progression. This would allow better determination of the specific risk factors predisposing survivors to cognitive decline in later years.

## Methods

### Study design and participants

A random subset of childhood ALL survivors was recruited from the St. Jude Lifetime Cohort Study to examine the impact of CRT on brain imaging outcomes. Survivors were eligible if they were treated with cranial radiation at St. Jude Children’s Research Hospital, were ≥10 years from cancer diagnosis and were ≥18 years of age. Participants were excluded if they developed a secondary cancer following additional cranial radiation treatment or non-treatment related CNS injury or disease prior to assessment. Of the 153 potentially eligible survivors, 127 were determined eligible for this study. Thirteen survivors refused participation, six withdrew from the study prior to completion of outcome measures and seven were excluded due to poor image quality, resulting in 101 MRI examinations of the brain for analysis. One participant with 716 microbleeds was a significant outlier and was excluded from the analyses. Specific chemotherapy regimens differed according to treatment era in this cohort. However, all survivors where treated with cranial radiation, vincristine, cyclophosphamide, 6-mercapitopurine, and methotrexate. Only two survivors did not receive asparaginase and only one survivor received daunomycin. This study was approved by the institutional review board at St. Jude Children’s Research Hospital, and all participants provided written informed consent to participate. All methods were performed in accordance with relevant guidelines and regulations.

### Neurocognitive testing

Neuropsychological testing was conducted within one day of brain imaging by a certified psychological examiner under the general supervision of a board-certified neuropsychologist. Primary neurocognitive functions assessed included intelligence (Wechsler Abbreviated Scale of Intelligence^21^), academics (Woodcock-Johnson III test of Achievement^22^), attention (Trail Making Test Part A^23^; Conners’ Continuous Performance Test-II^24^; Digit Span Forward from the Wechsler Adult Intelligence Scale III^25^), memory (California Verbal Learning Test-II^26^), processing speed (Grooved Pegboard^27^; Stroop Color Word Test^28^), and executive function (Trail Making Test part B^23^; controlled Oral Word Test; and Digit Span Backward^25^). Raw scores were transformed into age-adjusted z-scores based on population normative data. The Behavior Rating Inventory of Executive Function (BRIEF)^29^ was used to assess self-reported problems with inhibition, cognitive flexibility, emotional control, self-initiation, working memory, planning and organization, self-monitoring and organization of materials. BRIEF scores were reported as T-scores (μ = 50, σ = 10) based on reference to age and sex normative data.

### Image analysis

All imaging was obtained on a clinical 3 T MR scanner (Siemens Medical Solutions, Malvern, PA) and included a 3D T1-weighted MRI using sagittal 3D MPRAGE (magnetization prepared rapid acquisition gradient echo) sequence (TR/TE/TI = 1980/2.32/1100 ms) with an isotropic imaging resolution of 1.0 mm^3^. Axial susceptibility weighted imaging (SWI) sequences were obtained (TR 56 ms, TE 25 ms, Flip Angle 20°, slice thickness 2 mm, 0.55 mm in plane resolution) with reconstruction of phase and amplitude images performed on the scanner using Siemen’s Syngo SWI processing software.

Region of interest (ROI) selection was performed using an in-house developed program written in MATLAB (Math Works, Natick, MA). Two trained reviewers (NSP and JY) independently selected hypointense foci that were not contiguous with perpendicular blood vessels, flow voids or consistent with mineralization (calcification). During this analysis, small focal intracerebral hemorrhages only visible on susceptibility sensitive MRI sequences (cerebral microbleeds) and Zabramski cavernoma classification Type IV malformations were not differentiated as they are radiographically indistinguishable at 3 T and both are associated with radiation related small vessel abnormalities^30,31^. Selection of microbleeds was confirmed by a board certified neuroradiologist. All coding was conducted without knowledge of treatment exposure or neurocognitive function.

**Figure 1:**
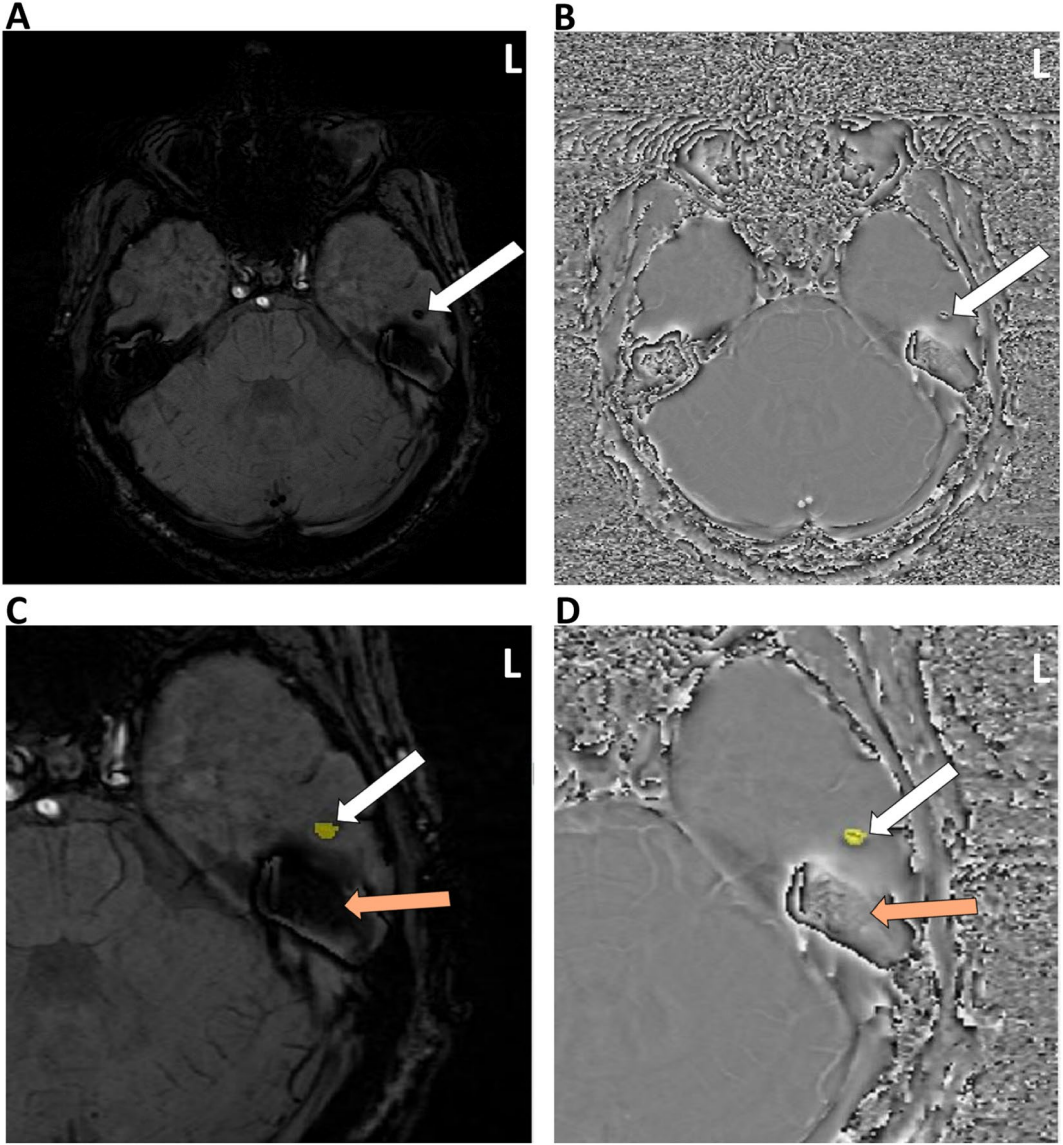
Example of a region of interest selection performed by the region filling algorithm. The arrow highlights the same microbleed in both the Susceptibility Weighted Minimum Intensity Projection image (SW mIP) (**A**,**C**) and corresponding Filtered Phase image. (**B**,**D**) Note detection and measurements could be accurately captured near areas of air induced susceptibility artifacts (orange arrow) using this method. (**C**,**D**) (Images are displayed in radiological convention).

Each microbleed was segmented using a 2D recursive region growing algorithm from a seed indicated by an experienced observer (Fig. 1). The connected 2D segments were combined to generate a 3D segmentation of the microbleeds. For each microbleed, the location of the center of mass and size in voxels and mm’s was recorded, as well as the microbleed count for each individual survivor. The coordinates for the center of mass of each microbleed were transformed into a standard brain reference space (MNI space) by first normalizing the SWI data set into template space using Statistical Parametric Mapping v12 (SPM 12)^32^. The resulting transformation was then applied to the coordinates of the center of mass. The coordinates were then mapped to Talirach space using the *icbm2tal* transform technique as previously described^33^. Location labels were generated for each microbleed based on the coordinates of the center of mass using the taxonomy maps developed by Brainmap.org.^34,35^. Microbleeds were assigned to one of the following locations: frontal lobe, parietal lobe, temporal lobe, occipital lobe, anterior lobe cerebellum, posterior lobe cerebellum, medulla oblongata, pons, sub-lobar (region defined to fill the remainder of volume within the hemisphere, such as the insular cortex) and midbrain. Locations were combined for left and right side. Additionally, microbleeds were categorized as located in grey matter or white matter, unless in the sub-lobar and brainstem regions where they were classified as mixed.

### Statistical analysis

Microbleeds were analyzed in two ways: presence of microbleeds for each brain region and cumulative microbleed counts (6 or more, top tertile vs other tertiles) due to a skewed distribution. Univariate analysis was conducted to identify neurocognitive scores significantly below the population mean (µ = 0, σ = 1.0) with those scores passing false discovery rate (FDR) correction selected for subsequent analysis. Multivariable modified Poisson regression was conducted to evaluate the effect of cranial radiation dose group (≥20 Gy vs <20 Gy) on the presence of microbleeds in selected brain regions, and multivariable linear regression was used to assess the effect of microbleeds (location and cumulative count) on neurocognitive outcomes without adjusting for multiple comparisons. All multivariable analyses were adjusted for age at diagnosis and sex. To further explore the effect of microbleed count on neurocognition, exploratory univariate analyses were conducted to compare the neurocognitive Z-scores between the 15 survivors who had no microbleed and the 15 people with 10 or more microbleeds. Additional exploratory analyses were conducted to evaluate if hypertension (a potential confounder) or methotrexate was associated with cumulative count of microbleeds. All analyses were conducted using SAS software version 9.4 (SAS Institute, Inc., Cary, North Carolina).

## Data Availability

The data and code that supports this research study are available from the corresponding author upon request. All data used in this study is available upon request. Please contact Kevin.Krull@stjude.org.
